# Enhancing kiwifruit flower pollination detection through frequency domain feature fusion: a novel approach to agricultural monitoring

**DOI:** 10.3389/fpls.2024.1415884

**Published:** 2024-07-25

**Authors:** Fei Pan, Mengdie Hu, Xuliang Duan, Boda Zhang, Pengjun Xiang, Lan Jia, Xiaoyu Zhao, Dawei He

**Affiliations:** ^1^ College of Information Engineering, Sichuan Agricultural University, Ya’an, China; ^2^ Ya’an Digital Agricultural Engineering Technology Research Center, Sichuan Agricultural University, Ya’an, China; ^3^ Agricultural Information Engineering Higher Institution Key Laboratory of Sichuan Province, Sichuan Agricultural University, Ya’an, China

**Keywords:** YOLO, frequency domain feature fusion, BRA, kiwifruit flower, precision agriculture deleted

## Abstract

The pollination process of kiwifruit flowers plays a crucial role in kiwifruit yield. Achieving accurate and rapid identification of the four stages of kiwifruit flowers is essential for enhancing pollination efficiency. In this study, to improve the efficiency of kiwifruit pollination, we propose a novel full-stage kiwifruit flower pollination detection algorithm named KIWI-YOLO, based on the fusion of frequency-domain features. Our algorithm leverages frequency-domain and spatial-domain information to improve recognition of contour-detailed features and integrates decision-making with contextual information. Additionally, we incorporate the Bi-Level Routing Attention (BRA) mechanism with C3 to enhance the algorithm’s focus on critical areas, resulting in accurate, lightweight, and fast detection. The algorithm achieves a $mAP_{0.5}$ of 91.6% with only 1.8M parameters, the AP of the Female class and the Male class reaches 95% and 93.5%, which is an improvement of 3.8%, 1.2%, and 6.2% compared with the original algorithm. Furthermore, the Recall and F1-score of the algorithm are enhanced by 5.5% and 3.1%, respectively. Moreover, our model demonstrates significant advantages in detection speed, taking only 0.016s to process an image. The experimental results show that the algorithmic model proposed in this study can better assist the pollination of kiwifruit in the process of precision agriculture production and help the development of the kiwifruit industry.

## 1 Introduction

Kiwifruit, one of the most widely consumed fruits globally, is prominent in the fruit market due to its distinctive characteristics and unique medicinal properties (Gökdoğan, 2022). Kiwifruit is a dioecious vine whose sex is recognized only at flowering and requires cross-pollination for fruiting. However, in practical orchards, natural biological pollination is often less efficient, which can adversely affect both fruit quality and the economic viability of production (Castro et al., 2021). Artificial pollination is commonly used to improve kiwifruit pollination rates in kiwifruit plantations. However, this method comes with high labor costs and the inability to accurately determine the success of pollination, leading to redundant work and increased planting expenses. In recent years, there has been rapid growth in precision agriculture and smart industry, with automated robots increasingly utilized across various agricultural domains (Yépez-Ponce et al., 2023), including robotic intelligent harvesting (Wang et al., 2022b), plant protection (Chen et al., 2021), and now, robotic pollination (Yang et al., 2023a). Robotic pollination is also expected to improve kiwifruit pollination efficiency and reduce labor costs (Gao et al., 2023a). The crux of this technology lies in accurately recognizing kiwifruit flower status and sex, particularly in complex orchard settings. Target detection, one of machine vision’s basic and challenging tasks, has been widely applied in agriculture by integrating deep learning techniques. These include fruit ripeness detection (Lu et al., 2022), pest and disease identification (Qi et al., 2022), weed detection (Hasan et al., 2021), fruit and vegetable quantity counting (Wang et al., 2022a), and pesticide residue detection (Liu et al., 2019). Deep learning and target detection techniques offer potential technical support for automating kiwifruit pollination.

Target detection algorithms based on deep learning mainly include two-step and one-step frameworks. Two-stage algorithms (e.g., the Faster R-CNN (Ren et al., 2017)) achieve object detection through candidate region generation and classification. However, they are computationally intensive and slow, limiting their applicability in real-world agricultural scenarios. In contrast, single-stage algorithms (e.g., the YOLO (Redmon et al., 2016)) extract features directly from the image for target classification and localization. This approach balances speed and accuracy, making it well-suited for real-time applications and capable of meeting industrial-grade detection standards. The advancement of these algorithms continues to drive forward computer vision technology, offering effective tools for a wide range of practical applications.

The YOLO algorithm stands out as one of the most popular methods in the field of target detection, widely adopted in artificial intelligence and computer vision for its speed and accuracy. In the domain of flower recognition, Wu (Wu et al., 2020) and colleagues developed a real-time apple blossom detection method based on YOLOv4 and CSPDarknet53 and optimized the model structure by channel pruning algorithm. The model reduces the number of parameters by 96.74% while maintaining high accuracy ( $mAP_{0.5}$ of 97.31%). However, their study primarily focused on detecting flower clusters, lacking a comprehensive exploration of extracting overall features of individual apple blossoms and failing to address detection challenges posed by fuzzy influences. Similarly, Xu et al. (Xu et al., 2022) introduced FlowerYolov5, a tomato bloom detection method based on the YOLOv5s architecture. This method incorporates a novel feature fusion layer. It integrates the Convolutional Block Attention Module (CBAM) to identify the bud, bloom, and fruiting stages of tomato flowers, leading to a notable improvement in $mAP_{0.5}$ by 7.8%, with a parameter count of 23.9M. However, the method neglected the detection of flowers in dense situations with a large number of parameters. Mithra (Mithra and Nagamalleswari, 2023) et al. employed UAV images to discern the sex of cucurbit plant flowers using deep transfer learning and a YOLOv4-based approach to support research on autonomous pollination. They manually labeled sex characteristics to identify and classify flowers in complex contexts, including interference, overlap, and ambiguous situations. This method achieves a $mAP_{0.5}$ of 91.2%, demonstrating its capability to quickly and effectively recognize small target objects in dense environments.

Researchers are also committed to identifying kiwifruit flowers to address the issue of autonomous pollination in kiwifruit. Williams et al. (Williams et al., 2020) first proposed a kiwifruit pollination robot and achieved an accuracy of 79.5% in flower recognition using convolutional neural networks. Lim et al. (Lim et al., 2020) utilized the Faster R-CNN Inception V2 model to achieve an accuracy of 91.9% in kiwifruit flower detection to enhance detection accuracy further. However, the Williams (Williams et al., 2020) and Lim et al. (Lim et al., 2020) teams only annotated female flowers, overlooking the potential occurrence of multi-class flowers during kiwifruit pollination. Consequently, Li et al. (Li et al., 2022b) emphasized the significance of identifying kiwifruit flowers and buds for robotic pollination, establishing a dataset that includes both flowers and buds. They achieved a $mAP_{0.5}$ of 91.49% with YOLOv4. Subsequently, in another study (Li et al., 2022a), the team introduced a novel method for multi-class detection and distribution recognition of kiwifruit flowers using YOLOv5l and Euclidean distance, further refining the classification of kiwifruit flowers. Their YOLOv5l model achieved a of 91.60%. In the agricultural sector, numerous researchers (Gai et al., 2023; Tian et al., 2023; Yu et al., 2023; Zeng et al., 2023) are refining the YOLO algorithm for the best performance in natural agricultural environments. Despite prior studies demonstrating commendable performance in detection accuracy, practical application issues such as model size and detection speed have been overlooked. The previously proposed models are larger and not applicable to real production environments.

Although the YOLO algorithm demonstrates advantages in target detection tasks, its performance in detecting dense targets within complex environments may be hindered by interference. The frequency domain features of the image contain essential information about the structure and content of the image, which helps to solve the dense detection problem of the YOLO algorithm in complex environments. Qin et al. (Qin et al., 2021) studied the channel attention problem by combining frequency domain features with a channel attention mechanism that compresses the channel using a customized Discrete Cosine Transform (DCT) but only utilizes a portion of each low-frequency message. Su et al. (Su et al., 2021) proposed a complete frequency channel attention network based on the attention mechanism in remote sensing. This approach enhances Qin et al.’s (Qin et al., 2021) weight acquisition method by attenuating high-frequency details and maximizing the utilization of low-frequency components. However, this network may lose some high-frequency details during noise filtering, leading to the omission of certain features. Duan et al. (Duan et al., 2023) introduced a detection method for infrared small targets integrating frequency-domain clutter suppression and spatial-domain feature extraction using deep learning techniques. They applied DCT to convert spatial-domain infrared images into the frequency domain. Following this transformation, the frequency domain information was filtered using an attention mechanism to adaptively enhance the target suppression background. Ultimately, they reversed the processed frequency-domain data to the spatial domain to facilitate feature extraction and fusion. The method was a lossy transform in which the image in the frequency domain does not retain all the details of the original image, resulting in the loss of important information. Through meticulous analysis and adept utilization of frequency domain information within an image, researchers can finely adjust feature extraction methodologies to accommodate diverse frequency ranges. This meticulous approach significantly enhances the precision with which critical image features are captured within deep learning frameworks. Nevertheless, the aforementioned methods do not fully exploit all features of the frequency domain and each incurs some degree of loss in frequency domain information.

Research models for flower recognition currently face challenges, especially in identifying kiwifruit flowers, such as oversized model dimensions and Ambiguous categorization of flower categories. This study proposes a lightweight algorithm called KIWI-YOLO in response to these challenges. KIWI-YOLO effectively reduces the parameters of the kiwifruit flower detection model and improves the accuracy of flower detection. The main contributions of this study are as follows:

(1) The kiwifruit flower’s whole stage image data set is constructed. In order to solve the problem of multi-category recognition of kiwifruit flowers in the natural environment, this study collected four kinds of kiwifruit flower image data covering spore, female flower, male flower, and pollination success state in real orchard environment. The original image is screened and labeled to form a full-stage image data set of kiwifruit flowers.
(2) A frequency domain feature fusion module is proposed. In order to improve the detection accuracy of the model in complex and dense environments, this study proposes a frequency domain feature fusion module (FDFF). This module enhances the model’s feature integration ability by extracting the high-frequency components of the original image and fusing them with the original image.
(3) BiFormerBlock is introduced to improve the model. In this study, by introducing BiFormerBlock into the C3 module of YOLOv5n, the perception ability of the model to fine-grained features is improved, and the accurate detection of smaller targets is realized.

Experimental data demonstrate that with 1,816,147 parameters, KIWI-YOLO achieves a $mAP_{0.5}$ of 91.6% and an F1-score of 86.5%. Compared to YOLOv5n, KIWI-YOLO shows an improvement of 3.8% in $mAP_{0.5}$ and 3.1% in F1-score. The experimental results show that KIWI-YOLO maintains a high recognition accuracy and significantly improves the feature expression ability on the basis of lightweight. It can quickly and accurately detect kiwifruit flowers and promote the further development of industrial automatic pollination technology.

## 2 Materials and methods

### 2.1 Data collection and processing

**Figure 1:**
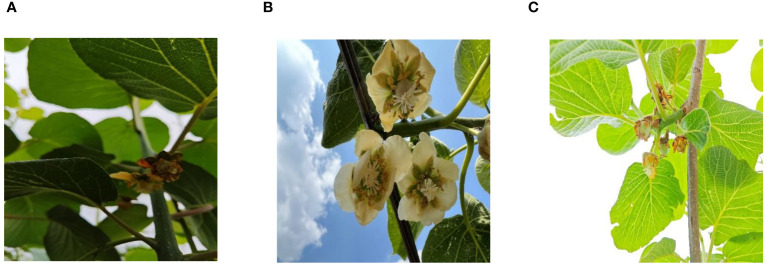
Examples of the different light. Panel **(A)** depicts the scenario under low light intensity, panel **(B)** demonstrates moderate light intensity, and panel **(C)** showcases high light intensity.

The experimentally collected data on all stages of the kiwifruit flower were obtained from Yucheng kiwifruit, a Chinese National Geographical Indication product, cultivated in Yucheng District, Ya’an City, Sichuan Province, China. This data was gathered during the 2023 flowering season in Longquan Village, Yucheng District, Ya’an City, Sichuan Province (103.03305, 30.00531). The angles of different growth states in the stage of kiwifruit flower and fruit are relatively consistent, all facing the ground. Therefore, when shooting, the shooting angle was from bottom to top in the vertical direction. The camera used was an iPhone 13 Pro Max with a resolution of 4032*3024, and data collection occurred under natural lighting conditions at 10:00 a.m., 2:00 p.m., and 6:00 p.m. The light during image acquisition includes low light, normal light, and overexposed high light to increase the richness of the data, as shown in **Figure 1**.

**Figure 2:**
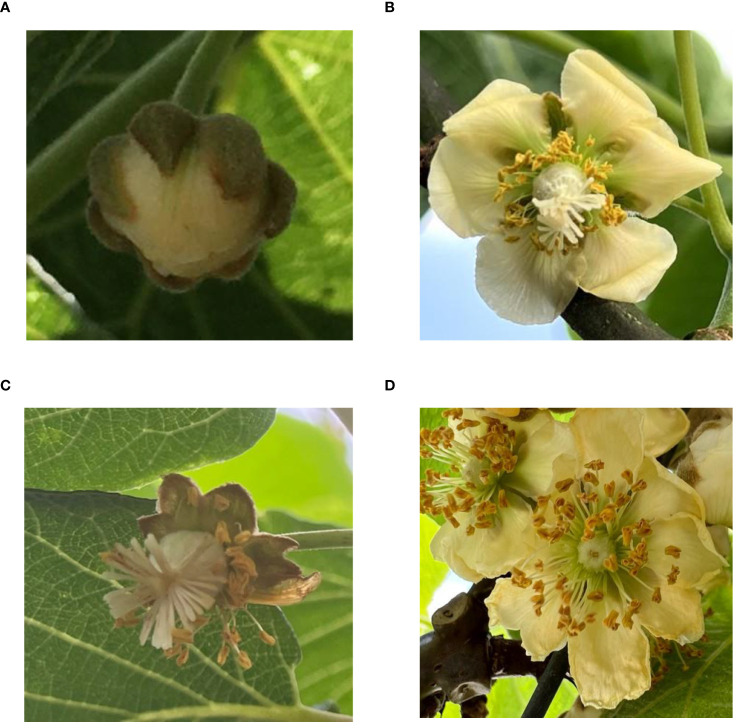
Four stages of kiwifruit flowers. Panel **(A)** is the bud stage of kiwifruit flower, panel **(B)** is the female flower of kiwifruit flower to be pollinated, panel **(C)** is the female flower of kiwifruit flower to be pollinated successfully, and panel **(D)** is the male flower of kiwifruit flower.

In the orchard environment, kiwifruit flowers, especially bracts, are usually densely distributed. The data set of this study covers such small and dense targets to ensure the effectiveness of the proposed algorithm in practical applications. The kiwifruit flower dataset consists of four stages: bud, female flower, male flower, and successful pollination, as depicted in **Figure 2**. The collection time of bud data was in early April 2023. Subsequently, according to the growth cycle of kiwifruit flowers, the collection time was from mid-April to mid-May. The data of male and female flowers were collected at the pollination stage after flowering, and the data of the pollination success category were collected one week after pollination. The dataset consists of 1594 images, which were divided into training sets and test sets at a ratio of 9: 1. Labelimg was used to manually annotate the collected data. The annotation file is in YOLO format. The total number of tags is 13450, covering the four characteristic classes of kiwifruit flowers, including Female (female flower), Bud (bud), Male (male flower), and Success (successful pollination state). Their distribution ratio is about 3:3:2:2.

Data augmentation was implemented on the collected data using an online approach to enhance the model’s robustness and generalization during training. Online enhancement preprocesses the data without altering the dataset size. In the online data augmentation approach, Mosaic (Bochkovskiy et al., 2020) randomly selects four images from the dataset, applies random cropping and scaling to each, and then arranges and stitches them together to create a single image. Mosaic enriches the dataset by adding small target samples. On the other hand, a mixup superimposes the two images according to their weights to generate a new image. Mixup makes the model focus more on the commonality of the data, which effectively improves the generalization ability and robustness of the model. This study combines Mosaic and Mixup to enhance the original data online.

### 2.2 YOLOv5 algorithm

The YOLOv5 architecture comprises three main components: Backbone, Neck, and Head. The Backbone aggregates image features across different scales using modules like Focus, BottleneckCSP, and SPP. It generates five feature layers from the input image, which are fused with feature maps from the Neck layer to enhance feature extraction. The Neck, adopting the FPN (Feature Pyramid Network)+PAN (Pixel Aggregation Network) structure, combines semantic and localization features. It integrates mixed image features and forwards them to the prediction layer. The Head is the classifier and regressor of YOLOv5. It determines the object corresponding to the feature based on the feature layer, recursively derives the target result of the detection, generates the bounding box, and predicts the category.

### 2.3 The proposed FDFF: frequency domain feature fusion module

In the whole stage of kiwifruit flower pollination, no matter whether it is the bud, female, male, or successful pollination kiwifruit flower state, the difference in color characteristics is small. When the neural network extracts the image features, the features provided by the color information are not enough to support the neural network in distinguishing the above four categories. In the actual production process, farmers distinguish the above four categories in the following ways:

(1) Shape: The bud exhibits a nearly circular shape, clearly distinct from the other three types.
(2) Presence of stigma: Distinguish male and female flowers by whether there is a stigma.
(3) Set fruit: Successful pollination of kiwifruit flowers is characterized by set fruit, and the main feature of this stage is whether the petals are in a state of shedding, and its shape is also significantly different from the other stages.

**Figure 3:**
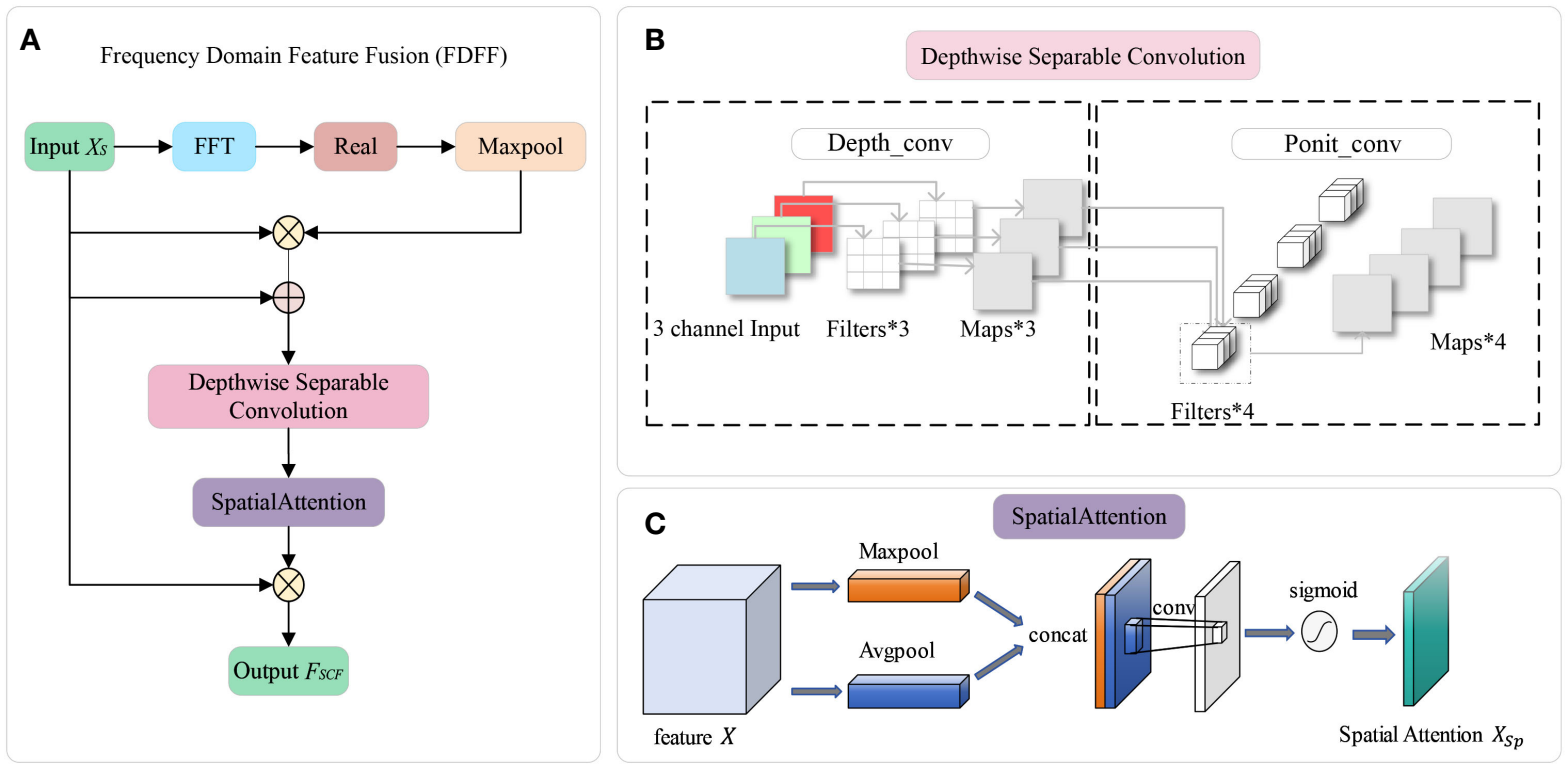
Panel **(A)** shows the overall flowchart of the FDFF module, which mainly includes the following modules: FFT layer, MaxPooling layer, DSC layer, and SpatialAttention layer. Panels **(B, C)** are the critical modules in FDFF. Panel **(B)** is the DSC structure diagram. Panel **(C)** is a spatial attention diagram.

The above three points can be summarized as the result of judging the kiwifruit flower by the contour information. In order to make the model extract the contour information more effectively, this study proposes a frequency domain feature fusion module (FDFF). FDFF transforms the feature map from the spatial domain to the frequency domain for processing and obtains the high-frequency component of the kiwifruit flower image (the high-frequency component correlates the part of the image with a faster gray change, videlicet the edge and contour of the image). The high-frequency component processed by the module is fused with the original feature map that retains the low-frequency component. After adding the FDFF module, the model can enhance the ability to obtain the contour information of each stage of kiwifruit flowers. The FDFF module consists of the Fast Fourier Transformation (FFT) layer, a MaxPooling layer, a Depthwise Separable Convolution (DSC) (Howard et al., 2017) layer, and a Spatial Attention (Woo et al., 2018) layer. The overall module flowchart of the FDFF module is shown in **Figure 3A**.

The FDFF module performs a two-dimensional Fourier transform of the input image through the FFT layer to convert the feature information in the spatial domain to the feature information in the frequency domain to extract the frequency domain features. Suppose the given tensor is $X_{S}\in R^{H\times W\times C}$ , which is converted to $X_{F}$ By FFT module. The conversion process is given by Equation 1:


(1) $$\begin{array}{c} X_{F}=\sum_{n=0}^{N-1}X_{s}e^{\frac{-j2\pi Fn}{N}},F=0,1,\ldots ,N-1 \end{array}$$

where *X_s_* denotes spatial-domain feature information, $X_{F}$ denotes frequency-domain feature information, and j denotes the imaginary part; F denotes the subscript of the frequency-domain sampling point, ranging from 0 to N-1; and n denotes the subscript of the spatial-domain sampling point, ranging from 0 to N-1.

A two-dimensional complex matrix is obtained by FFT, with the real part representing the magnitude of the information in the frequency domain and the imaginary part representing the phase information. In this study, the improvement effect of the frequency domain data model is determined by comparing the use of only the real part, only the imaginary part, and the combination of the real part and the imaginary part. The experimental results show that the $mAP_{0.5}$ using only the real part is 91%, using only the imaginary part is 88.9%, and using both the real and imaginary parts is 90.01%. It is speculated that the high-frequency component corresponds to the important features of the image, and the amplitude is invariant. However, the interpretation and utilization of phase is more complex, the model is difficult to understand and use the relevant information, and the performance enhancement is limited. The task of this study is more important for the perception of global structure and texture. Therefore, this study uses the amplitude and spectrum and ignores the phase information by taking the real part of the operation inside the FDFF, which simplifies the problem and improves efficiency.

The max-pooling layer in this module aims to achieve frequency domain filtering and reduce the dimensionality of the data. The max-pooling selects the maximum value in each local region in the frequency domain to extract the main high-frequency features, which help identify the salient frequency domain components in the image, including texture features.

After the max-pooling layer operation, the output feature map *X_M_* is obtained. Before the data enters the DSC layer, these frequency domain components are fused in the frequency domain to strengthen the frequency domain feature information. The multiplication of *X_M_* with the original input data *X_S_* is performed as shown in Equation 2.


(2) $$\begin{array}{c} X_{MS}=X_{M}*X_{S} \end{array}$$

where *X_MS_* is the output.

After completing the fusion of frequency domain components, the FDFF module splices the obtained feature map containing high-frequency contour information with the input feature map to ensure the integrity of the spatial-frequency domain feature information so that the model can make full use of the relevant features. In order to reduce the complexity of the algorithm, the FDFF module uses DSC to perform feature fusion and tensor dimension transformation on the obtained feature map. DSC divides the convolution operation into two sub-operations: Depthwise Convolution and Pointwise Convolution. Compared with ordinary convolution, DSC has the advantages of small parameters and small computation, which can improve the speed of convolution operation.

As shown in **Figure 3B**, Depth_conv uses a convolution kernel for each channel separately, and the output channel of a single channel is one after the convolution operation. For the three-channel RGB map, Depth_conv outputs three feature maps with channel 1. Then, the three feature maps are sequentially spliced to get an output feature map with channel N. Through Ponit_conv processing, the convolution kernel is 1*1*M(M is the number of channels in the previous layer). Based on the previous map, a new feature map is generated by a weighted combination in the depth direction, which is used to reduce the dimension of the input feature map while maintaining the feature information’s invariance. The purpose of independent convolutional computation for each channel is to reduce the number of parameters, reduce the computational effort, and increase the computational efficiency. The number of parameters for conventional convolution is Equation 3, and the number of parameters for DSC is Equation 4. The comparison of the formulas shows that the number of parameters and computation of the depth separable convolution is one-third of the conventional convolution.


(3) $$\begin{array}{c} parameter_{conv}=W_{F}\times H_{F}\times C_{I}\times C_{O} \end{array}$$

(4) $$\begin{array}{c} parameter_{dsc}=W_{F}\times H_{F}\times C_{I}+1\times 1\times C_{I}\times C_{O} \end{array}$$

where $W_{F}$ is the width of the convolution kernel, ${\text{H}}_{\text{F}}$ is the height of the convolution kernel, $C_{I}$ is the number of input channels and $C_{O}$ is the number of output channels.

The role of the spatial attention layer is to allow the task network to focus more on finding helpful information related to the current output to improve the quality of the output. Using spatial attention can act on the pixels of the feature map to weigh each pixel and fully use each pixel’s information, as shown in **Figure 3C**. After the weights of spatial attention have been extracted, they are multiplied by the original input $X_{S}$ . At this point, the spatial attention will focus more on the position of the kiwi flower to be recognized in the picture, and then the feature map $F_{SCF}$ is obtained.

### 2.4 C3BiF based on dynamic sparse attention mechanism

**Figure 4:**
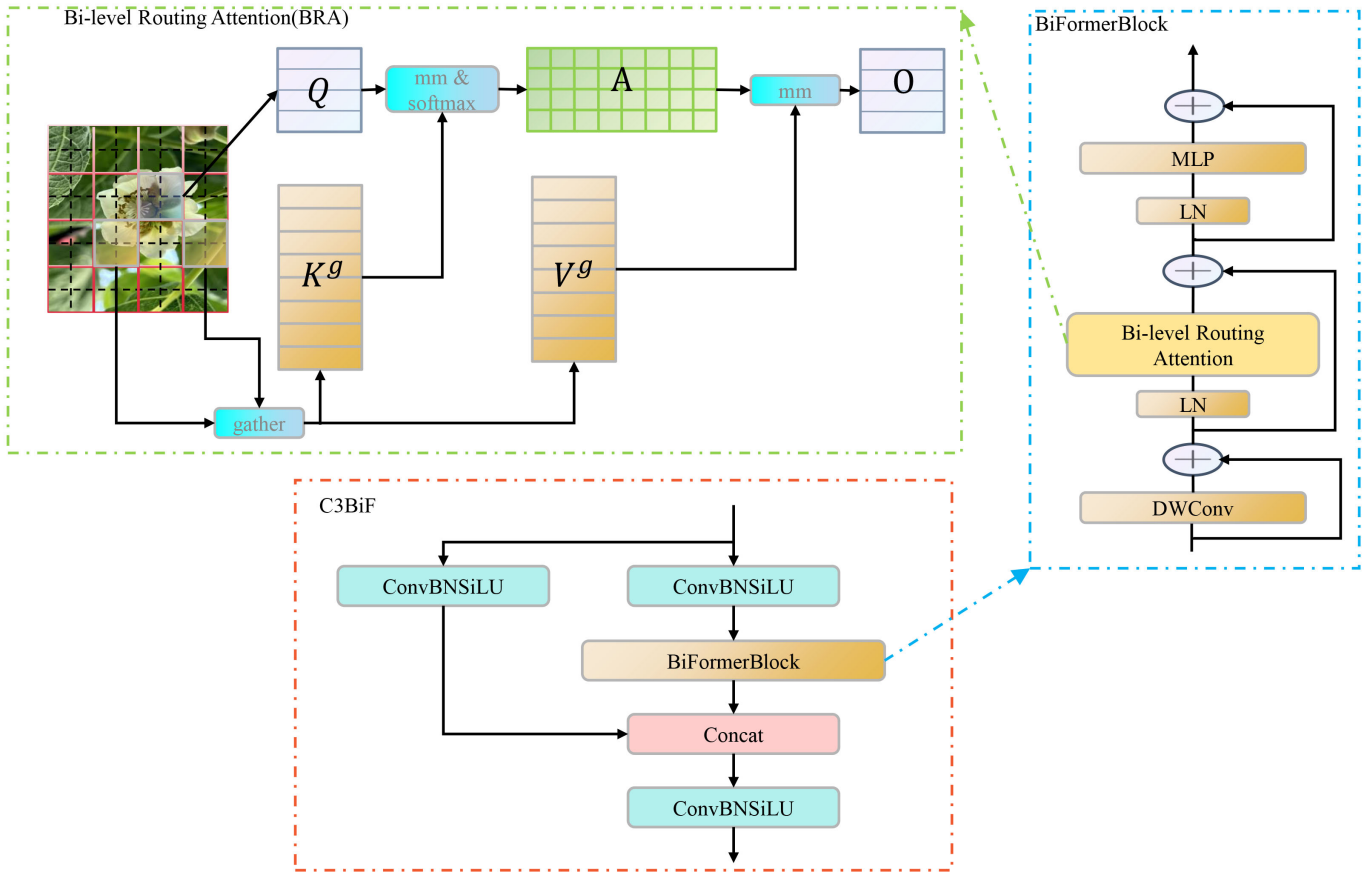
Components of the overall C3BIF architecture. BRA is the core of the C3BIF module. In the architecture of the BRA mechanism, Q is used to compute the keywords that are weighted in relevance to the query. K denotes the keyword or identifier used to provide information or for matching purposes. V is the value associated with the query result and the keyword information. A is the adjacency matrix denoting the semantic relevance between two regions. O is the output of the attention mechanism.

The attention mechanism aids the model in focusing on the most relevant parts of the input data, thereby capturing key information and establishing correlations between inputs more efficiently. In this study, the C3BiF module is proposed to replace part of the C3 modules in YOLOv5 for feature extraction. The C3BiF module introduces a BiFormerBlock module containing Bi-level Routing Attention (Zhu et al., 2023) by utilizing the constructor of the C3 module, as shown in **Figure 4**. The module computes attention in two stages: first, coarse-grained attention is performed. The image is divided into blocks to control sparsity, and self-attention is performed at this level. Inter-block correlations are computed using Q and K to form a relation matrix A. Next, A is sparsified, retaining the largest elements and identifying pairs of blocks that require further attention. Finally, based on the sparse matrix from the first stage, fine-grained self-attention is performed, and each patch only performs the attention computation on patches in other blocks related to its block.

The calculation of attention mechanism (Woo et al., 2018; Hu et al., 2018; Wang et al., 2020) weights requires the joint participation of the target and all elements, which is the attention mechanism between input and output. VIT (Dosovitskiy et al., 2020) uses the self-attention mechanism (Vaswani et al., 2017) to capture long-term dependencies for the problem of establishing the correlation between multiple inputs in the visual field. However, the self-attention mechanism often imposes a substantial computational burden and memory overhead. In this paper, BRA is used to address the above application bottlenecks. BRA leverages sparsity to reduce computation and memory usage, enabling dense matrix multiplication suitable for GPUs. This approach demonstrates excellent performance and computational efficiency, particularly in dense prediction tasks. A series of studies (Gao et al., 2023b; Yang et al., 2023b; Zhang et al., 2023) have indicated that, since BRA is based on sparse sampling rather than downsampling, it can retain fine-grained detail information. So, the BiFormerBlock containing the BRA module is suitable for detecting small objects.

### 2.5 The improved KIWI-YOLO algorithm

Both YOLOv8n and YOLOv5n are considered excellent nano models. In the preliminary experiments of this study, the number of parameters was 1764577 for YOLOv5n and 3302624 for YOLOv8n. The number of parameters for YOLOv8n was 1.8 times that of YOLOv5n, and $mAP_{0.5}$ was only improved by 0.03 points. Compared with the exponential increase in the number of parameters, the detection performance does not increase exponentially but leads to an increase in model complexity. Considering the subsequent application architectures of the study, the lightweight variant of YOLOv5, known as YOLOv5n, optimized for Nanodevices, has been chosen. It provides detection accuracy suitable for edge devices while maintaining high speed. The model has a channel number factor of 0.25 and a depth factor of 0.33.

**Figure 5:**
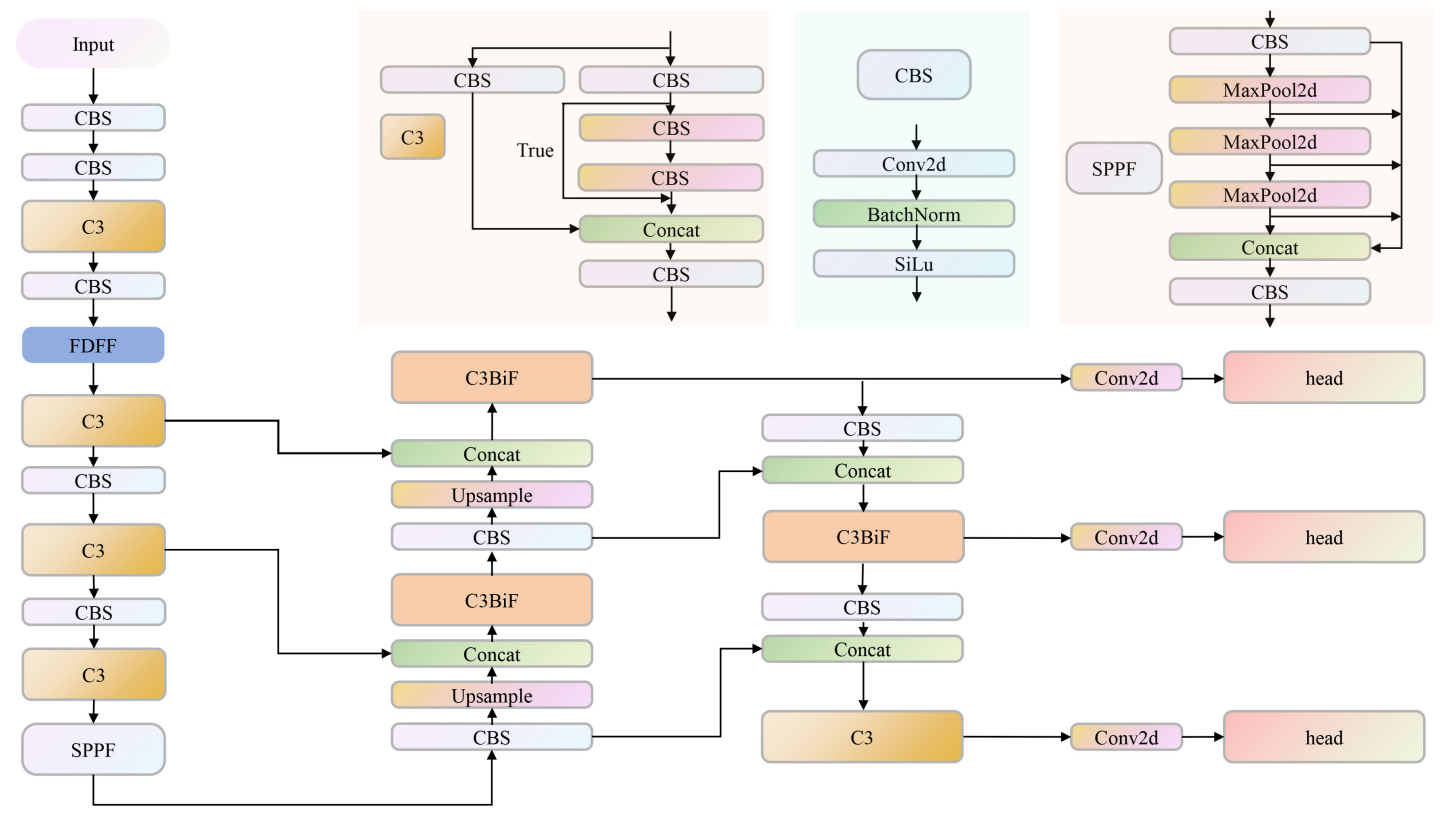
The structure of the KIWI-YOLO model. KIWI-YOLO comprises CBS, C3, SPPF, FDFF, C3BIF, and other modules. CBS encapsulates the combination of convolution, batch normalization, and activation function. C3 contains multiple CBS modules and a Bottleneck module. SFFP is a fast spatial pyramid pooling containing multiple maximum pooling layers and CBS modules. FDFF and C3BIF are the innovative modules proposed in this study.

This study was based on the improvement of the YOLOv5n network structure. In order to improve the detection accuracy of the algorithm, the fourth layer of the network (the next layer of P3) was replaced by the FDFF structure mentioned in the article. The FDFF improves the ability to extract and express network features. In addition, the overall detection efficiency is affected by the presence of small targets during the whole stage of pollination of kiwifruit flowers. Therefore, C3BiF containing BiFormer structure was introduced in the Head part. The overall KIWI-YOLO algorithm model is shown in **Figure 5**.

### 2.6 Training environment and evaluation indicators

In this study, the hardware was NVIDIA Quadro RTX 5000 Graphics Processing Unit (GPU) with 16GB of graphics memory and 128GB of RAM, and the Central Processing Unit (CPU) was i9-10900K, 3.7GHz. The system was Windows Server 2019 Standard, and PyCharm built the network environment with the deep learning framework PyTorch 2.0.0, the Python version of which was 3.8. For training, all images were uniformly resized to 640*640. The batch size for input images during training was set to 16, and the number of training rounds was 300. The optimizer employed was the SGD optimizer to optimize the network parameters. The initial learning rate (lr0) was set to 0.01, with an initial learning rate momentum of 0.937 and a weight decay of 0.0005.

The YOLO algorithm assesses its object detection system based on two core metrics: detection accuracy speed and detection accuracy. Detection speed refers to the time taken to detect one image. Detection accuracy is evaluated using metrics such as Precision (P), Recall (R), F1-score, Average Precision (AP), and mean Average Precision (mAP), calculated as described in Equations 5–9. In target detection, Intersection over Union (IoU) quantifies accuracy by comparing detection overlap and true labeled boxes. Typically, a ratio greater than 0.5 indicates confident detection. Moreover, the value of mean average precision, denoted as $mAP_{0.5}$ , calculated at IoU = 0.5, is crucial for assessing the overall detection accuracy of the model. A higher $mAP_{0.5}$ signifies better detection accuracy.


(5) $$\begin{array}{c} Precision=\frac{TP}{TP+FP}\times 100\% \end{array}$$

(6) $$\begin{array}{c} Recall=\frac{TP}{TP+FN}\times 100\% \end{array}$$

(7) $$\begin{array}{c} F1=\frac{2\times Precision\times Recall}{Precision+Recall} \end{array}$$

(8) $$\begin{array}{c} AP=\int_{0}^{1}P\left(R\right)dR \end{array}$$

(9) $$\begin{array}{c} mAP=\frac{1}{M}\sum_{m=1}^{M}AP_{m} \end{array}$$

In the above Equation, TP denotes True Positive, TN denotes True Negative, FP denotes False Positive, FN denotes False Negative, M is the number of target classes to be detected, and $AP_{m}$ is the average precision of the mth class of targets.

## 3 Experiments and results

### 3.1 FDFF module analysis

In this study, the insertion position of FDFF was experimentally compared to confirm the optimal insertion position of the FDFF module. The role of FDFF is to extract and fuse the features, so it is considered to be inserted into the backbone part of YOLOv5n to enhance the performance of the model’s multilevel feature extraction. FDFF is inserted into the $P_{n}+1$ layer respectively for comparative experiments to find the best effect. The experimental findings are summarized in **Table 1**. According to the experimental results, when the FDFF insertion is $P_{3}+1$ , the effect is the best, the parameter is 1790195, the $mAP_{0.5}$ reaches 91%, and the $mAP_{0.5}$ is 3.2% higher than that of the non-insertion. The increase of $mAP_{0.5}$ inserted into the later layer of P1, P2, P4 and P5 was 2.5%, 2.7%, 2.7% and 2.5% respectively.

**Table 1:** Results of FDFF module experiments; [*P*_n_] corresponds to the nth Conv layer in the YOLOv5 backbone feature extraction network.

FDFF Adds Location	$mAP_{0.5}$ (%)	Recall(%)	Parameters	Time(s)
Base	87.8	78.4	1764577	0.013
$P_{1}+$ 1	90.3(+2.5)	82.6(+4.2)	1766899	0.015
$P_{2}+$ 1	90.5(+2.7)	82.7(+4.3)	1772707	0.027
$P_{3}+1$	**91.0(+3.2)**	**83.6(+5.2)**	1790195	0.016
$P_{4}+1$	90.5(+2.7)	84.6(+6.2)	1882819	0.013
$P_{5}+$ 1	90.3(+2.5)	82.1(+3.7)	2230339	0.016

$P_{n}+1$ corresponds to the next layer of $P_{n}$ .The bold values in the table emphasize the experimental results of the methods used in this study.

Analyzing the FDFF module, it can be seen that the module obtains the high-frequency information in the frequency domain while retaining the low-frequency information, fuses the frequency domain with the spatial domain, and strengthens the contour information of the kiwifruit flower image. The shallow layer of the feature extraction network mainly learns some relatively simple features, which are not abstract and complex enough for the task. In the deeper layers of the network, the neural network can learn more abstract and complex features, but at the same time, it faces the problems of training difficulty and gradient. FDFF performs better in the middle layer, which makes it easier for the gradient to be propagated in the network. In this position, it can better balance the abstraction, computational efficiency, and training effect.

### 3.2 C3BIF module analysis

In this study, in order to demonstrate the effect of the number of C3BIFs and the position of adding them on the network results, experiments were conducted on models that have been added with the FDFF module. **Table 2** presents the experimental results. As can be seen from the table, the best effect is achieved when the first three C3 layers of the Head layer are all replaced with C3BIF and the $mAP_{0.5}$ reaches 91.6%, which is a 0.6% growth compared to the model with no replacements, and the number of parameters is 1816147. However, the growth effect of other replacement schemes is not obvious, and it will even cause a decrease of $mAP_{0.5}$ .

**Table 2:** Results of C3BIF module experiments.

N	$mAP_{0.5}$ (%)	Recall(%)	Parameters	Time(s)
1	90.9	82.3	1801395	0.018
2	90.3(-0.6)	83.4(+1.1)	1793747	0.013
3	90.6(-0.3)	82.7(+0.4)	1801395	0.012
1、2	91.0(+0.1)	83.5(+1.2)	1804947	0.018
2、3	90.1(-0.8)	83.2(+0.9)	1804947	0.017
1、3	91.1(+0.2)	82.3(+0.0)	1812595	0.014
**1、2、3**	**91.6(+0.7)**	**83.9(+1.6)**	**1816147**	**0.016**

N represents the Nth C3 in the Head layer and is replaced with C3BIF.The bold values in the table emphasize the experimental results of the methods used in this study.

Analyzing the C3BIF module, it is observed to contain the BiFormerBlock featuring Bi-level Routing Attention, which exhibits enhanced recognition capabilities for smaller targets. Since the fourth C3 in the head layer primarily deals with smaller feature maps for detecting large-scale targets, the foremost requirement for enhancement lies within the first three C3 layers of the Head. These initial C3 layers contribute significantly to the detection of small and medium-scale targets. Therefore, replacing three initial C3 layers with C3BIFs at the same time produces better results.

### 3.3 Ablation experiments of the KIWI-YOLO network

**Figure 6:**
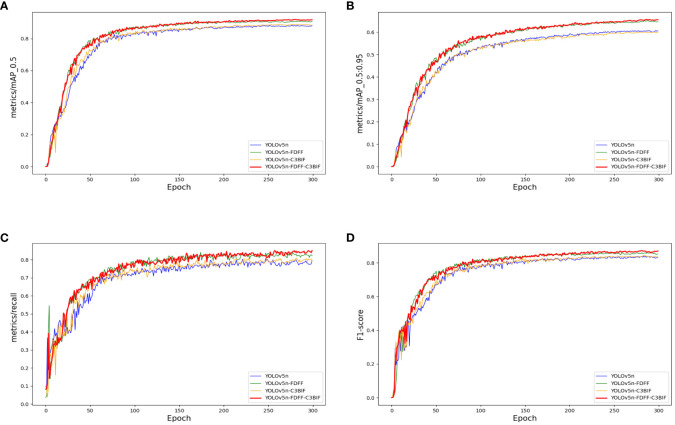
Comparison of ablation experimental results. **(A)**. Panels **(A-D)** are the result curves of *mAP*_0.5_, *mAP*_0.5:0.95_, recall and F1-score, respectively.

Ablation experiments were conducted on each module to assess the effectiveness of the enhanced KIWI-YOLO network model in kiwifruit flower detection. The experimental results are summarized in **Table 3**, and the visualization curve is shown in **Figure 6**.

**Table 3:** Results of ablation experiments.

Baseline	FDFF	C3BIF	$mAP_{0.5}$ (%)	$mAP_{0.5:0.95}$ (%)	Recall (%)	F1-score (%)	Parameters	Time (s)
**YOLOv5n**			87.8	60.7	78.4	83.4	1764577	0.013
	√		90.8(+3.0)	65.1(+4.4)	82.2(+3.8)	86.1(+2.7)	1790195	0.016
		√	88.6(+0.8)	60.0(-0.7)	79.4(+1.0)	83.7(+0.3)	1790529	0.021
	**√**	**√**	**91.6(+3.8)**	**65.8(+5.1)**	**83.9(+5.5)**	**86.5(+3.1)**	**1816147**	**0.016**

$mAP_{0.5}$ , $mAP_{0.5:0.95}$ , Recall, F1-score, parameter size, and detection time for ablation experiments with the improved model.The bold values in the table emphasize the experimental results of the methods used in this study.

As depicted in **Table 3**, the utilization of the FDFF module alone yields a significant improvement in mAP, with $mAP_{0.5}$ surging by 3 points, surpassing the 90% mark. Furthermore, $mAP_{0.5:0.95}$ experiences an increase of nearly 5 points. The Recall and F1 parameters also exhibit a notable improvement of approximately 3 points each. Remarkably, the detection time per image only marginally increases by 0.003 seconds. When solely incorporating the C3BIF module into the original YOLOv5 network, the enhancement is evident but not as pronounced. However, when the FDFF module is combined with the C3BIF module, $mAP_{0.5}$ notably rises to 91.6%, marking a 3.8-point increase compared to the base network. Similarly, Recall improves to 83.9%, reflecting a 5.5-point enhancement. Despite this improvement, the increase in the number of parameters remains minimal. Additionally, the detection time per image also improves by 0.003 seconds. These findings underscore the significant detection enhancements achieved by the improved network at a minimal additional cost.

### 3.4 Multi-category recognition accuracy analysis

The model distinguishes among four growth stages of kiwifruit flowers that occur during kiwifruit flower pollination. The average accuracies of the improved model were 95%, 89.6%, 93.5%, and 88.2% for the four categories: Female, Bud, Male, and Success, respectively. Compared to the original YOLOv5n network, the improvements were 1.7%, 3.9%, 6.2%, and 3.2%, respectively. A comparison of the results is presented in **Table 4**.

**Table 4:** Multi-category recognition effect.

Classification	Original YOLOv5	Improved model
Female	93.3%	**95.0%(+1.7)**
Bud	85.7%	**89.6%(+3.9)**
Male	87.3%	**93.5%(+6.2)**
Success	85.0%	**88.2%(+3.2)**

The average accuracy of the four categories Female, Bud, Male, and Success on the original YOLOv5 network and the improved KIWI-YOLO, respectively. The bold values in the table emphasize the experimental results of the methods used in this study.

**Figure 7:**
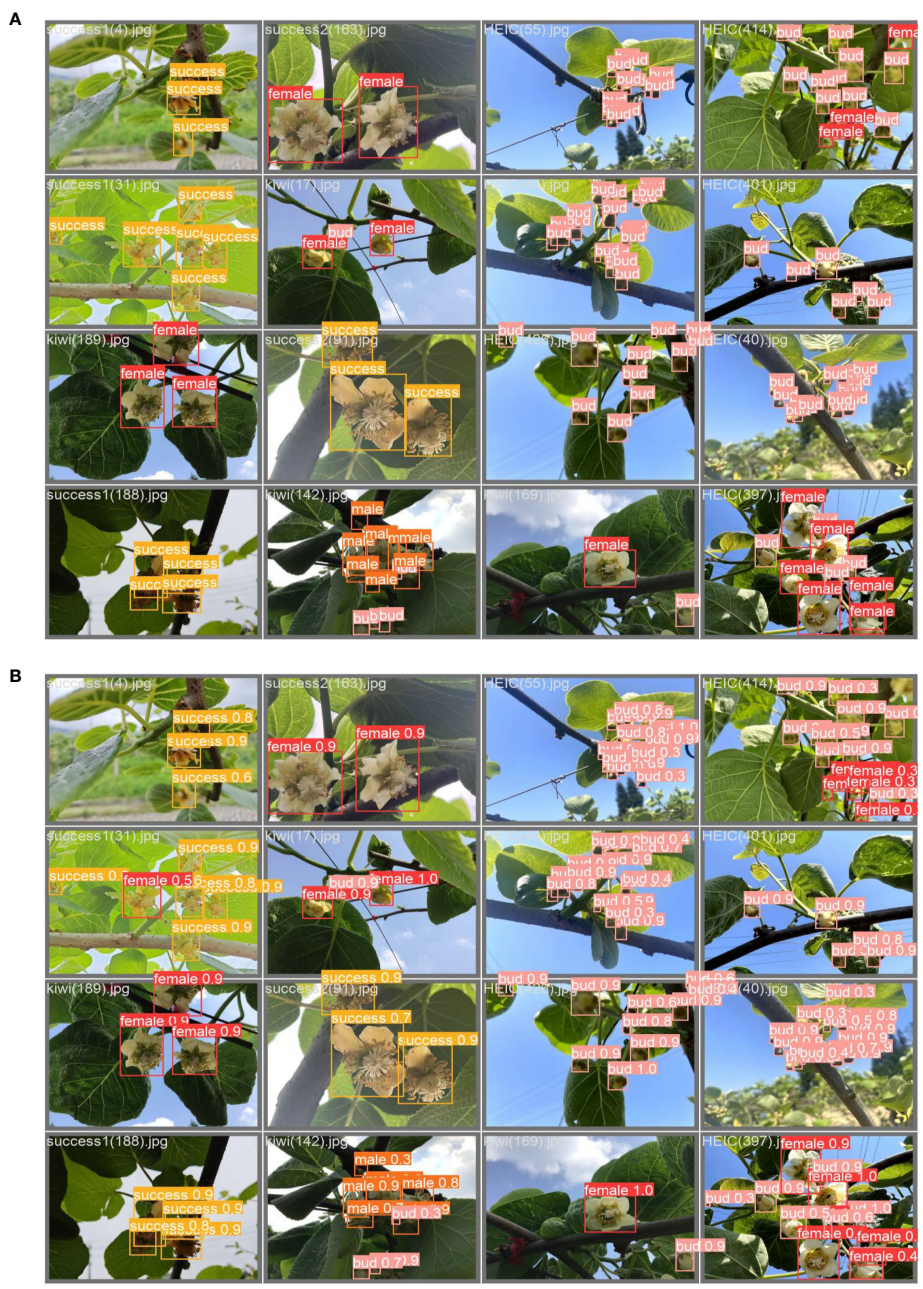
Detection effect diagram: Panel **(A)** shows the real label, and panel **(B)** shows the predicted label, i.e., the model detection result.

The enhanced model exhibited more noticeable improvements in detecting Bud and Male stages compared to Female and Success. Nevertheless, the average detection accuracy exceeded 93% for both the Female and Male categories. The successful class represents the successfully pollinated kiwifruit flowers, which is challenging for this type of detection. This difficulty arises from the slight morphological differences between this class and Female in the early stages of successful pollination, making them hard to distinguish. Furthermore, in the later stages, when the petals are fully removed, the morphology closely resembles that of Female without petals, resulting in a slightly weaker detection performance. **Figure 7** illustrates the detection efficacy under various conditions, including different types, shading scenarios, and variations in lighting intensity.

### 3.5 Mainstream algorithm comparison

To evaluate the effectiveness of the KIWI-YOLO model proposed in this paper for detecting all stages of kiwifruit flower pollination, we compared its performance with current mainstream target detection models. The chosen algorithms include the two-stage target detection model Faster R-CNN (Ren et al., 2017), the one-stage target detection model SSD (Liu et al., 2016), YOLOv5s, YOLOv5n, YOLOv8s, and YOLOv8n, as well as the excellent lightweight target detection models at this stage, YOLOX-nano (Ge et al., 2021), YOLOX-tiny (Ge et al., 2021), and YOLOv7- tiny (Wang et al., 2023). The performance comparison of these detection models is shown in **Table 5**.

**Table 5:** Results of mainstream algorithm comparison experiment.

Model	$mAP_{0.5}$ (%)	F1-score(%)	Parameters	Average precision for each category (%)			
				Female	Bud	Male	Success
Faster R-CNN	70.12	62.25	137098724	81.28	55.87	71.56	71.77
SSD	81.05	78.00	26285486	83.26	77.97	85.31	77.66
YOLOX-nano	79.12	76.00	912159	81.61	78.27	81.47	75.12
YOLOX-tiny	83.91	76.34	5033739	84.94	83.26	87.69	79.75
YOLOv5n	87.80	83.49	1764577	93.30	85.70	87.30	85.00
YOLOv5s	90.70	86.14	7020913	94.80	88.70	91.60	87.90
YOLOv7-tiny	89.20	86.09	6015714	93.90	86.10	90.90	85.70
YOLOv8n	88.10	83.13	3302624	92.80	87.30	88.40	83.80
YOLOv8s	89.70	85.94	11127132	92.90	88.40	91.40	86.00
**KIWI-YOLO**	**91.60**	**86.50**	**1816147**	**95.00**	**89.60**	**93.50**	**88.20**

$mAP_{0.5}$ , F1-score, number of parameters, and average accuracy of each category for ten models on the kiwifruit flower dataset.The bold values in the table emphasize the experimental results of the methods used in this study.

Through analyzing the test results in this table, it can be concluded that the $mAP_{0.5}$ of the KIWI-YOLO model proposed in this paper is higher than these several detection models, 21.48, and 10.55 percentage points higher than Faster R-CNN, SSD. Compared to the current popular one-stage models, it is 3.8, 0.9, 2.4, 1.9 and 3.5 percentage points higher than YOLOv5n, YOLOv5s, YOLOv7-tiny, YOLOv8s and YOLOv8n, respectively. Compared to YOLOv5s, the superiority in $mAP_{0.5}$ is small, but in the model’s size, it can be seen that KIWI-YOLO reduces the number of parameters by almost 75% compared to YOLOv5s. In comparing YOLOv5n, although the number of parameters increased by 0.05M in model parameters, it increased by 3.8% in $mAP_{0.5}$ and 3.01% in F1-score. For the lightweight YOLOX model, KIWI-YOLO is weaker than YOLOX-nano in terms of the advantage of the number of parameters, but $mAP_{0.5}$ improves by 12.48 percentage points and by 7.69 percentage points over YOLOX-tiny.

**Figure 8:**
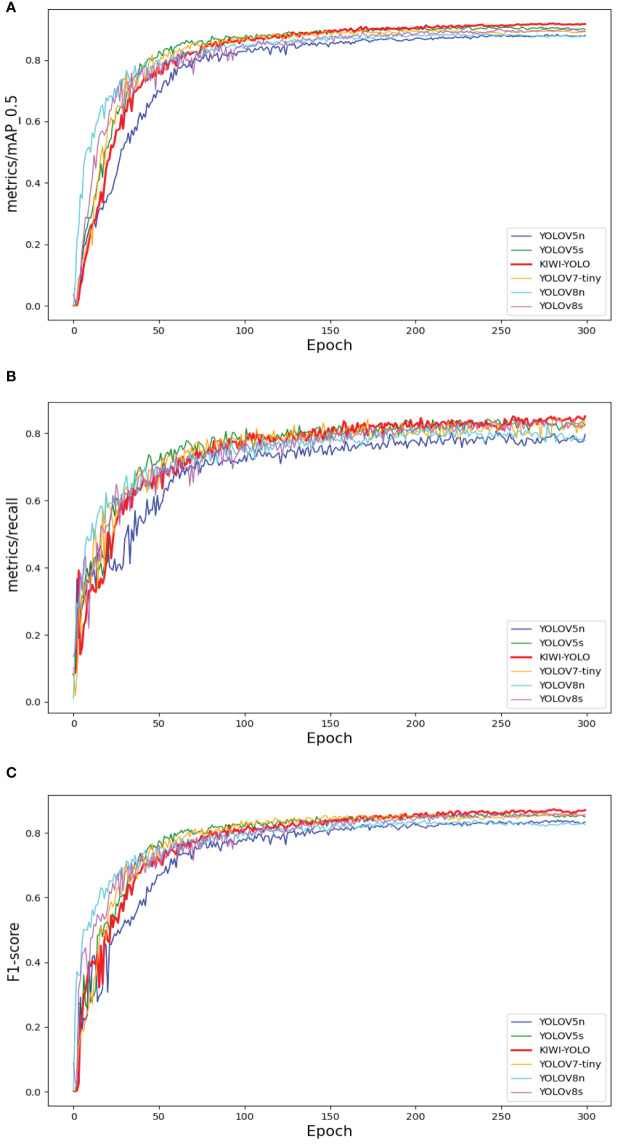
Mainstream Algorithm Performance Comparison. Panel **(A)** compares the average precision curves, **(B)** compares the recall, and **(C)** compares the F1-score.

Among them, the excellent one-stage target detection algorithms YOLOv5n, YOLOv5s, YOLOv7-tiny, YOLOv8s, and YOLOv8n at the present stage are selected and compared with the KIWI-YOLO algorithm in this study and their average accuracy curves and Recall curves are compared with the line graphs as shown in **Figure 8**.

The experimental results show that the improved model KIWI-YOLO proposed in this study has a certain degree of superiority in terms of comprehensive performance compared with the currently popular target detection models. The model can accurately and quickly realize the detection task of kiwifruit pollination in all stages. In addition, the model can basically meet the requirements of embedded devices in terms of size and is lightweight.

### 3.6 The Grad-CAM analysis

**Figure 9:**
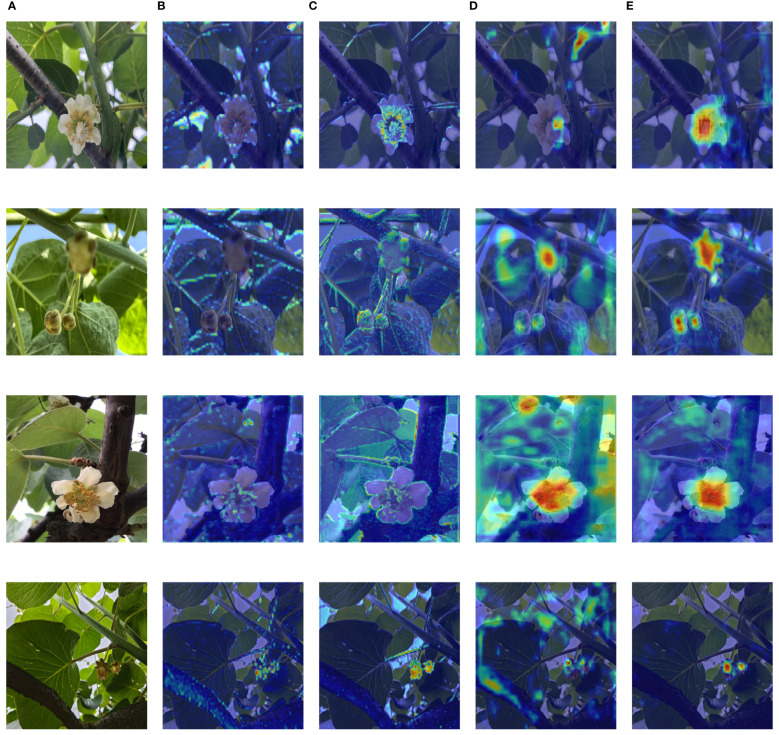
Comparison of heat maps. The four rows represent Female, Bud, Male, and Success. Panel **(A)** is the original image. Panel **(B)** is the heat map of the original P4 layer output. Panel **(C)** is the heat map output after adding FDFF. Panel **(D)** is the heat map of the original C3 output. Panel **(E)** is the heat map of the added C3BIF output.

Grad-CAM (Selvaraju et al., 2017) can visualize the part of the neural network that contributes the most to the prediction result. In this paper, the decision-making process of the neural network is visualized by the Grad-CAM technique, and the help of the improved module on the detection task is illustrated. **Figure 9** displays original images of four different categories alongside heat maps generated for each improved key layer using Grad-CAM. In these heat maps, the redder the color, the more the region contributes to the model results.

The heat map in **Figure 9** shows that through the frequency domain feature fusion in the FDFF module, the network model gives more importance to the target contour but also pays attention to the overall contour of the branches and leaves. This is due to the overall frequency domain features of the image being extracted in the FDFF module, and the context information in the frequency domain is obtained by fusion with the global frequency domain features. From the comparison between columns B and C, it can be seen that the model with FDFF can combine the global frequency domain information, make full use of the context information to understand the relationship between the environment and the detection target, and better identify the target. Observing the D and E columns of **Figure 9**, it can be seen that before optimization by the C3BIF module, the focus of the network is more dispersed, and there are still some red areas that represent the focus of attention outside the target. After optimization with the C3BIF module, the network is more focused on the target. However, in complex environments, the model still remains weakly focused on the blade due to the similarity of the blade and target colors. From the heat map, the improved KIWI-YOLO model has achieved a more accurate detection of the four flower states during the whole stage of kiwifruit flower pollination.

## 4 Discussion

At present, in the field of kiwifruit flower recognition, researchers aim to improve the recognition accuracy of related models without considering the actual situation of agricultural environment. Most of the models have problems such as fuzzy categories (Lim et al., 2020; Williams et al., 2020) and excessive size (Li et al., 2022a), which limit the effective application of the model on embedded devices. In this paper, we summarize the current problems of kiwifruit flower recognition into the following three points:(1)The classification of kiwifruit flowers is fuzzy. (2)Existing model size is too large. (3)Small size model accuracy is low. Therefore, this paper has carried out relevant research on these three issues.

In this study, the dataset was manually collected and labeled to solve the fuzzy problem of kiwifruit flower classification. Based on the actual situation of the kiwifruit pollination period, the flowers are mainly divided into four categories: Female(female flower), Male(male flower), Bud(bud), and Success(successful pollination). Aiming to address the problem of the model size being too large, the existing mainstream models are compared. The two-stage detection models, such as Faster R-CNN (Ren et al., 2017) and SSD (Liu et al., 2016), require large memory and are inaccurate in localization. In the one-stage detection model, YOLOv7 (Wang et al., 2023) introduces an efficient layer aggregation network to improve performance. However, it performs poorly on the small target detection task and shows weak generalization ability. As a new product of the YOLO series, YOLOv8 has a complex model structure, and its size is too large to be suitable for embedded devices. YOLOX (Ge et al., 2021) has an advantage in the number of parameters, but performed mediocrely on the kiwifruit flower recognition task. In contrast, YOLOv5 introduces CSPDarknet53 as the backbone network and utilizes the PANet(Path Aggregation Network) structure to enhance feature fusion, demonstrating good accuracy and speed. The KIWI-YOLO model proposed in this paper is based on the lightweight YOLOv5n algorithm, which meets the requirements of embedded devices for model size and maintains speed and accuracy. In this study, the FDFF module is proposed to solve the problem of low recognition accuracy of small-size models. In kiwifruit cultivation, farmers usually distinguish between different types of flowers by observing their morphological characteristics. YOLOv5n has shortcomings in extracting contour information, and the $mAP_{0.5}$ in the experiment is only 87.80%. The FDFF module can extract high-frequency information and fuse spatial domain information, avoid the omission of important information, and improve the lossy process of information extraction in previous studies (Qin et al., 2021; Su et al., 2021; Duan et al., 2023). After adding the FDFF module, the $mAP_{0.5}$ of the model is increased by 3%. In addition, the bud characteristics are significantly different from those of the other three types of kiwifruit flowers, while the accuracy of YOLOv5n was still low, only 85.7%. This study further proposes a C3BIF module combined with the BRA mechanism. The module enhances the recognition accuracy of the model for small targets and improves the detection accuracy of the Bud by 3.9%.

The KIWI-YOLO algorithm proposed in this study shows significant superiority over existing methods. Compared to the baseline model, it achieved a 3.8% improvement in the overall $mAP_{0.5}$ and a 3% improvement in the F1-score, which fully demonstrated its effectiveness. In addition, the number of model parameters is only 1.8M, the average accuracy is as high as 91.6%, and it takes only 0.016 seconds to complete the processing of an image. This high efficiency makes the KIWI-YOLO model very suitable for deployment in automated systems. The model can accurately identify the flowers in the pollination period, thus significantly reducing invalid pollination attempts and saving manpower and resources. Its accurate detection results provide real-time data and accurate pollination timing for orchard managers, avoiding failure and fruit quality decline caused by premature or late pollination. It helps to optimize the production process and resource allocation, improve the efficiency and sustainability of agricultural production, and maintain the stability and health of the orchard ecosystem.

This study has made preliminary progress in improving the classification accuracy of kiwifruit flowers, but there are still a series of challenges. (1) The accuracy of the model in recognizing the Bud and the Success targets is not satisfactory. This is mainly because Bud targets are smaller, denser, and easily overlap. On the other hand, the Success and the Female have similar features and often appear in the same scene, which makes the model prone to misidentification. In the future, this study will continue to solve the difficult problem of small target recognition, explore the addition of a small target detection head in KIWI-YOLO, add a lightweight upsampling CARAF operator, and so on. (2) In terms of the dataset, this study collected images with different light and different shading situations to increase the richness of the data but did not conduct separate experiments for light classification and experiments with different shading situations. At the same time, the image data failed to cover the whole environment of the actual orchard, and the generalization of the model needs to be improved. To this end, future work will focus on collecting more comprehensive data and conducting specialized classification experiments on illumination and occlusion effects. (3) Existing images contain noise and sharpening problems due to the limitation of acquisition equipment. It is planned to replace professional equipment such as robots or drones to obtain higher-quality image data. (4) Currently, more and more researchers use multi-modal data for object detection. Subsequent studies will consider collecting multi-modal data on kiwifruit and test the fusion effect of the FDFF module on modal data.

## Data availability statement

The raw data supporting the conclusions of this article will be made available by the authors, without undue reservation.

## Author contributions

FP: Conceptualization, Writing – original draft, Writing – review & editing. MH: Data curation, Validation, Writing – original draft, Writing – review & editing. XD: Funding acquisition, Writing – review & editing. BZ: Data curation, Validation, Writing – review & editing. PX: Data curation, Writing – review & editing. LJ: Writing – review & editing. XZ: Supervision, Writing – review & editing. DH: Data curation, Writing – review & editing.
